# Applying a Social Exclusion Framework to Explore the Relationship Between Sudden Unexpected Deaths in Infancy (SUDI) and Social Vulnerability

**DOI:** 10.3389/fpubh.2020.563573

**Published:** 2020-10-20

**Authors:** Rebecca A. Shipstone, Jeanine Young, Lauren Kearney, John M. D. Thompson

**Affiliations:** ^1^School of Nursing, Midwifery, and Paramedicine, University of the Sunshine Coast, Sippy Downs, QLD, Australia; ^2^Departments of Paediatrics, Child and Youth Health, and Obstetrics and Gynaecology, Faculty of Medical and Health Science, University of Auckland, Auckland, New Zealand

**Keywords:** sudden unexpected deaths in infancy (SUDI), Sudden Infant Death Syndrome—SIDS, disadvantage, social exclusión, deprivation, social vulnerability and vulnerable

## Abstract

**Background:** Sudden Unexpected Death in Infancy (SUDI) is a leading cause of preventable infant mortality and strongly associated with social adversity. While this has been noted over many decades, most previous studies have used single economic markers in social disadvantage analyses. To date there have been no previous attempts to analyze the cumulative effect of multiple adversities in combination on SUDI risk.

**Methods:** Based on sociological theories of social exclusion, a multidimensional framework capable of producing an overall measure of family-level social vulnerability was developed, accounting for both increasing disadvantage with increasing prevalence among family members and effect of family structures. This framework was applied retrospectively to all cases of SUDI that occurred in Queensland between 2010 and 2014. Additionally, an exploratory factor analysis was performed to investigate whether differing “types” of vulnerability could be identified.

**Results:** Increased family vulnerability was associated with four major known risk factors for sudden infant death: smoking, surface sharing, not-breastfeeding and use of excess bedding. However, families with lower levels of social vulnerability were more likely to display two major risk factors: prone infant sleep position and not room-sharing. There was a significant positive relationship between family vulnerability and the cumulative total of risk factors. Exploratory factor analysis identified three distinct vulnerability types (chaotic lifestyle, socioeconomic and psychosocial); the first two were associated with presence of major SUDI risk factors. Indigenous infants had significantly higher family vulnerability scores than non-Indigenous families.

**Conclusion:** A multidimensional measure that captures adversity across a range of indicators highlights the need for proportionate universalism to reduce the stalled rates of sudden infant death. In addition to information campaigns continuing to promote the importance of the back-sleeping position and close infant-caregiver proximity, socially vulnerable families should be a priority population for individually tailored or community based multi-model approaches.

## Introduction

In Australia, Sudden Unexpected Death in Infancy (SUDI) is the leading category of potentially avoidable infant mortality and a significant public health concern. It is a term used to describe the sudden and unexpected death of a seemingly well infant aged <1 year, in which there is no immediately apparent cause of death, and includes Sudden Infant Death Syndrome (SIDS) as well as deaths subsequently explained following a thorough investigation. Risk factors for SUDI have been extensively described, and include low birth weight, preterm birth, pre- and post-natal tobacco smoke exposure, not breastfeeding, prone and side sleep position, sleeping in a separate room from caregivers, soft or cushioned surfaces, loose bedding, and certain forms of surface sharing (1).

Social factors play a notable role in SUDI. A significant association between SUDI and social disadvantage has been reported, with the association strengthening in more recent years (2). While the public health campaigns of the 1990s—which advised parents to place their infants supine to sleep—saw SUDI in Australia decline by over 85% (3), over the past two decades rates have plateaued and the social gradient widened. Although need for SUDI studies to more thoroughly investigate infants' social environments using a combination of different markers of disadvantage has long been recognized (4), most studies have continued to use single markers of social position in analysis of disadvantage. Where a combination of factors has been identified, the overwhelming approach has been to adjust for their influence, rather than to try to understand the cumulative effect of multiple factors in combination. It has recently been recommended that multiple adversities be analyzed simultaneously to more fully understand their influence on health outcomes (5). It has also been suggested that a “sociological turn” away from a purely medical paradigm for understanding SUDI is needed to more fully explore the interaction between SUDI and social disadvantage (6, 7).

Running parallel to health sciences within which SUDI literature is published is a field of sociological enquiry focused on the measurement of disadvantage. Within this field, social exclusion has emerged as a concept to describe an accumulation of disadvantage, including lack of resources, participation and skills (8). The present study represents a unique endeavor to adapt and apply a social exclusion framework to investigate the link between SUDI and multiple adversities.

## Background: Social Exclusion Theory and Research

Although there is no universally accepted definition of social exclusion, there is consensus that social exclusion is characterized by certain key features. At its core, social exclusion is multidimensional (it implies disadvantage across a wide range of indicators) and relational (it implies a major discontinuity in the relationship of the individual with the rest of society). It is a dynamic process driven by an interplay of interconnected demographic, economic and social factors (8, 9). This disadvantage is cumulative, often resulting in the intergenerational transfer of disadvantage (10). A synthesis of the major frameworks is presented in Supplementary Table 1.

The extent of social exclusion varies between individuals and families; some will exhibit exclusion on few or no indicators and are therefore considered less vulnerable than people experiencing multiple forms of exclusion. More important than social exclusion at a particular point in time is persistent social exclusion (10). In order to measure the *extent* of social exclusion, data is most appropriately collected at the individual, family or household level rather than at geographic area or societal level (11, 12). Identifying *persistence* also requires longitudinal data. Consequently, the data requirements of such frameworks are high. Data availability is a well-documented obstacle to the measurement of social exclusion.

To date the most comprehensive studies estimating the population prevalence of social exclusion have used measures derived from longitudinal social surveys (5, 11, 13) or the Census (14). However, characteristics associated with social exclusion such as having a marginal socioeconomic position, low education and literacy, and high mobility are also risk factors for attrition of longitudinal study participants (15) as well as Census non-participation (16). Longitudinal studies have difficulty in maintaining representative samples of socially vulnerable populations and are likely to underestimate the extent of disadvantage and adversity in a population. They are also suboptimal for studying lower prevalence health outcomes; in a large longitudinal study of 5,000 infants, only 10 cases of SUDI would occur given current rates. Similarly, since the Census is conducted periodically and is not linked to administrative data in most countries, Census data has limited utility in examining health outcomes.

### Social Exclusion Among Infants and Young Children

Individual level indexes have issues of applicability across the age spectrum; indicators chosen to measure social exclusion therefore vary according to lifecycle stage (10). Since infants and young children are inextricably bound to and dependent on their families, child social exclusion reflects the marginal positions of the family unit (17). Studies of child social exclusion usually include variables to measure characteristics of children's parents, families and households (17, 18).

### Family and Household Level Measures of Social Exclusion and Adversity

There are few previous efforts to develop a multidimensional measure of household or family level disadvantage: the Australian Bureau of Statistics used binary indicators from the Socioeconomic Indexes for Areas (SEIFA) (19, 20), and more recently the 2016 Census (14) on which families and/or households scored positively for each indicator displayed by one *or more* member. This method neither accounts for the increasing disadvantage experienced with the increasing prevalence of certain indicators within a household, nor the potential effect of family structures. This was an acknowledged limitation, with the methodological complexity of such a project noted.

Scutella, Wilkins and Kostenko (13) constructed a measure of household exclusion defined as the *average exclusion* scores of all individual “adult” members aged over 15 years. However, designating children aged over 15 years as adults is problematic. Applying this approach, a sole parent with five school-age children (two of whom are over 15 years), who experienced mental ill-health, would be scored as having a *lower* degree of social exclusion than a couple-parented family with one young child, in which only one parent experienced mental ill-health. Using averages when assigning a vulnerability score to caregivers is an effective means of accounting for the role that certain family structures play in producing or limiting vulnerability, while simultaneously accounting for the increasing disadvantage that occurs when more than one caregiver has a vulnerability characteristic. However, the average exclusion method is not effective at identifying increasing disadvantage with corresponding prevalence among siblings. Siblings do not have the same potential as adults to ameliorate the effect of a vulnerability characteristic but may exacerbate it.

Gubhaju et al. (5) identified indicators of family adversity in the Longitudinal Study of Australian Children and applied exploratory factor analysis to develop 12 dimensions and two higher order spheres of adversity. This approach accounted for the effect of family structures by including a five-item construct of disadvantage, linked to family background (sole parent, stepparent, step or half siblings, four or more children). An additional score (0–4) was assigned to the overall adversity score, depending on family characteristics. Binary indicators were used for all variables and as such this method did not account for increasing disadvantage with increasing prevalence among family members.

Each approach has strengths and limitations. Consequently, a range of techniques was adapted to develop a framework by which to measure family-level social vulnerability in the Queensland SUDI Study. The term social vulnerability is used in this study in preference to “disadvantaged” or “excluded.” Here it refers to the potential inability of families to withstand adverse impacts from multiple stressors to which they are exposed. However, since vulnerability can be understood as the counterpart to resilience, it simultaneously acknowledges people's capacity to adapt in the face of challenging circumstances. It also recognizes the important role of structural factors in creating and maintaining disadvantage.

## Aims

The overarching aim of this study was to develop a multidimensional framework capable of producing an overall measure of the level of social vulnerability among families who experienced a SUDI. More specifically, the aim was to produce a family-level index, which recognizes that social vulnerability may increase in severity with both the increasing prevalence of certain indicators within a family and their persistence over time, while at the same time taking account of family structure. The secondary aims were to investigate whether there were differences in the causes of death and epidemiological factors among families with greater social vulnerability compared to less vulnerable families; whether distinct “types” of social vulnerability could be identified; and whether a relationship existed between particular epidemiological factors and specific vulnerability “types.”

This is a unique effort to explore the relationship between infant mortality and social vulnerability in Australia. No previous studies could be found that examined the relationship between family-level social vulnerability and risk factors for sudden infant death.

## Methods

### Study Context

This study was part of a broader retrospective cohort study of all cases of SUDI in Queensland, Australia between 2010 and 2014 (the Queensland SUDI Study). In Queensland, all cases of SUDI are required to be reported to a Coroner. Once an infant is discovered unexpectedly deceased, police and coronial investigations commence. Police officers interview the family and collect information relating to the infant's sleep environment, recent medical history, and the family's social, child protection and criminal history. The process of, and information recorded during, investigation has been reported in detail previously (21–23).

### Data Collection

Indicators of social vulnerability were identified using information available in data routinely collected when infants and their families have contact with health or child protection services, and during a coronial death investigation. Documents analyzed in relation to each death included the Police Report of Death to a Coroner (containing narrative descriptions of the circumstances of death including the social and familial context), witness statements, autopsy reports, coronial findings, and child protection records. There is a degree of subjectivity in collecting data from SUDI reports, which contain substantial amounts of narrative text. The formats of these documents differ, data come from different time periods and the data collection leaves room for interpretation. Consequently, both the intra-rater and inter-rater reliability of data was assessed on a number of variables, which found excellent levels of agreement (kappa > 0.8). Cases of SIDS and unexplained sudden infant death (USID) were classified according to the currently accepted San Diego definition (24). Due to known inconsistences in cause of death certification, all SUDI cases were re-classified by an expert review panel, the details of which have been reported elsewhere (22). The study investigated infant care practices, infant sleeping arrangements, antenatal, postnatal, and recent clinical history, social circumstances and living situation. Specific information about the collection and recording of a number of variables that are not straightforward in definition have been previously published (23). Detailed information regarding study cases, screening, and data collection have been reported elsewhere (23). SUDI cases were linked to the Perinatal Data Collection, the Queensland Hospital Admitted Patient Data Collection, and the Emergency Department Information System. Data linkage methods and linkage rates for each data source have been previously reported (25). Study data were collected and managed using Research Electronic Data Capture (REDCap), a secure, web-based application designed to support data capture for research studies. Ethical approval was provided by the University of the Sunshine Coast Human Research Ethics Committee (HREC: S/15/805).

### Development of a Social Vulnerability Framework and Score

A two-step process was employed to construct the framework for identifying family-level social vulnerability. First the literature on social exclusion and disadvantage was used to identify appropriate indicators of family vulnerability (see Supplementary Table 2). For clarity, since this framework proposes the indicators that *ought* to be used, this has been termed the Normative Social Vulnerability Framework (SVF). In total, 38 indicators of family-level social vulnerability were identified. Step two involved the development of a more restricted set of measures capable of application to the Queensland SUDI Study dataset. After consideration of the data, the initial list of indicators was reduced to a final list of 32 variables. Table 1 describes indicators in both the Normative and the Queensland SUDI Study SVF.

**Table 1:** Normative and Queensland SUDI Study Social Vulnerability Framework indicators by family-weighted composite score method.

	**Normative Social Vulnerability Indicators**	**Queensland SUDI Study Indicators**	**Family-weighted composite score method**
Income and material resources	**Family income below poverty line** Family has Australian Government healthcare/concession card		
	**Limited material resources and financial hardship**^a^ In past 12 months any of: Couldn't afford to buy groceries or clothing Couldn't pay rent/mortgage Pawned/sold possessions Assistance from welfare organizations	Proxy measure due to data availability. Any of: Insufficient food in home Inadequate food/clothing for children Infant fed cow's milk/powder as alternative to formula Difficulty paying rent/mortgage or problems securing accommodation Poor housing quality, lack of amenities, housing filthy, infestations Inability to afford other essentials (e.g., medications, whitegoods)	Binary
Employment	**Under and precarious employment** Neither parent full-time, one only part-time, casual, or unstable work	Proxy measure due to data availability.Low status parental occupations^b^ ( ≤ 25th percentile).	Continuous measure 1 (lowest)-−0.25
	**Unemployment** Neither parent employed <12 months	Both parents recorded as ‘unemployed' or no occupation listed on birth/death records	Binary
	**Long-term unemployment** Neither parent employed ≥12 months	Long-term parental unemployment noted in case records	Binary
Education and skills	**Early school leaver** Parents didn't complete high school		
	**Very early school leaver** Parents didn't complete junior high school		
	**No advanced or vocational education** Parents have no formal education beyond high school and no vocational training		
	**Poor basic skills** Parents have poor literacy/English competence		
Housing	**Homelessness** Sleeping rough, couch surfing, homeless shelters	Recent history of homelessness	Binary
	**Marginal homelessness** Insecure accommodation e.g., boarding houses, caravans, makeshift dwellings	Recent history of insecure forms of accommodation	Binary
	**Overcrowding** Australian Bureau of Statistics definition based on number of bedrooms and relationships between residents in dwelling^c^	Any of: Multiple families in one residence Entire family sharing one room (no other space available), living areas functioning as bedrooms More than 2 children sharing a bedroom, or pubescent children of opposite sex sharing a bedroom	Binary
	**Transience and mobility** Multiple moves during pregnancy during infant's life	Either of: >2 changes of address during pregnancy and after birth Transience noted in case records	Binary Longitudinal indicator
	**Public housing** Living in public/community housing		
Health and healthcare	**Chronic illness, condition or disability**	Parent/s have a chronic illness, condition or disability	Weighted for family type and increasing prevalence
		One or more siblings has a chronic illness, condition or disability	Weighted for family type and increasing prevalence
	**Mental ill-health**	Mental ill-health in parents	Weighted for family type and increasing prevalence
	**Alcohol misuse**	Recent alcohol misuse history by parents	Weighted for family type and increasing prevalence
	**Substance use**	Recent substance abuse history or drug diversion therapy by parents	Weighted for family type and increasing prevalence
	**Infant and child mortality**	Previous liveborn sibling death	Binary
		Previous stillbirth	Weighted for increasing prevalence
	**Multiple miscarriages or terminations**	Multiple terminations or miscarriages	Weighted for increasing prevalence
	**Limited access to primary care**	Data available for mother and infant only. Any of: Hospitalization for ambulatory care-sensitive conditions (ACSC)^d^ Emergency presentation for primary or dental care Missed appointments (including did not wait) Infant failure to thrive Recurrent parasitic infections in infant or siblings Inadequate antenatal care e	Weighted for increasing prevalence
Crime and threats to safety	**Criminal offending**	Parents charged with criminal offense <12 months	Weighted for family type and increasing prevalence
	**Long term criminal offending**	Parents consistent criminal offending in adulthood (≥12 months)	Weighted for family type and increasing prevalence Longitudinal indicator
	**Victim of crime**	Parents victim of crime <12 months	Weighted for family type and increasing prevalence
	**Domestic and family violence**	Domestic violence present in current or previous relationships	Binary Longitudinal indicator
	**Child abuse and neglect** Child in family experienced or at risk of physical, sexual or emotional abuse or neglect	Infant or sibling known to child protection services ≤ 3 years	Binary
	**Engagement in risky behaviors**	Parent/s currently or previously engaged in sex work	Binary Longitudinal indicator
Transport and access to services	**Could not access services due to a lack of transport**	Any of: Difficulty running the family car (i.e., fuel, registration) Difficulty attending medical appointments or important events due to a lack of transport Difficulties accessing public transport	Binary
	**Does not have access to car in household**		
	**Geographic isolation** Geographic distance imposes a high restriction upon accessibility to goods, services and opportunities for interaction	Living in an area where Accessibility and Remoteness Index of Australia (ARIA+)^f^ classification = remote or very remote or outer regional	Binary Weighted for remoteness classification
Family and social relationships	**Limited social support** Mother's social support index (condensed)^g^	Proxy measure due to data availability. Parent/s experience any of: Fractious and unsupportive familial relationships Involuntary separation of family due to incarceration of partner or partner working away without extended family support	Binary
	**Multiple partners** Parents have children to multiple different partners	Parents have one or more children to one or more previous partners	Weighted for family type and increasing prevalence
	**Child not in the care of biological parents**	Children in informal care arrangements and/or children placed in care of child protection agencies	Binary Longitudinal indicator
	**Stressful life events** Life events index (condensed)^h^	Proxy measure due to data availability. In previous 12 months ≤ 2 of: Tumultuous parental and extended family relationships Recent separation, divorce or separation/reconciliation pattern Recent re-partnering Child custody disputes Recent change in care arrangements Recent imprisonment or court appearance Death of a family member Recent move Recent job loss or demotion Major change in health of a family member	Weighted for increasing prevalence
Intergenerational disadvantage	Parental history of child abuse and neglect	Parents known to child protection services as children	Weighted for family type and increasing prevalence
	Parental history of juvenile offending	Parents history of juvenile offending noted in case records	Weighted for family type and increasing prevalence

= N/A in Queensland SUDI Study dataset.

a Financial hardship indicators are an abridged version of the hardship items used in Australian longitudinal population-based surveys (26, 27).

b The Australian Socioeconomic Index 2006 (AUSEI06) was applied to parental occupation data coded using the Australian and New Zealand Standard Classification of Occupations (28). The higher of the two occupational statuses represented the family's occupational status.

c (29).

d ACSCs are avoidable with the application of public health interventions and early disease management, delivered in a primary care setting and are a marker for access to timely and effective primary care (17).

e The Kotelchuck Adequacy of Prenatal Care Utilization Index was used to categorize antenatal care as “adequate,” “intermediate,” or “inadequate” based on gestation at entry into antenatal care, number of visits and gestation at delivery (30).

f The Accessibility/Remoteness Index of Australia (ARIA+) divides Australia into classes of remoteness based on relative access to services (31).

g Standardized tool for measuring maternal social support (32).

h *Standardized tool for measuring stressful life events (33)*.

The 32 Queensland SUDI Study SVF variables were first used in a binary (yes/no) manner, such that a family were assigned the value 1 if *any* member of the immediate family displayed the characteristic, to create a simple summation vulnerability score with a possible score range of 0–32. This is in line with the “sum-score” approach used by Scutella, Wilkins and Kostenko (13) and the Australian Bureau of Statistics' method for creating a multidimensional family-level measure of social disadvantage (19).

A family-weighted composite measure was also constructed to account for (i) increasing severity of vulnerability with increasing prevalence of certain indicators within a family, (ii) potential effect of family structures, and (iii) persistence of certain indicators of vulnerability over time. Construction of the family-weighted composite score proceeded from three basic premises. Premise one entails that when more than one parent and/or more than one child within a family experience a characteristic the level of vulnerability is increased. For example, if both parents in a couple-parented family experience mental ill-health the family is likely to have a greater degree of social vulnerability on that indicator than if only one parent experiences the condition. While it was not possible to accommodate all family structures, premise two holds that the degree of vulnerability experienced by a single, unsupported parent is likely to bear close similarity to that of a couple-parented family in which both parents display a characteristic. Premise three is that families who experience persistent disadvantage over time are likely to be more socially vulnerable than those experiencing disadvantage at a specific point in time. These premises are underpinned by extensive research; the link between single parenthood and adverse child health outcomes has been repeatedly demonstrated (34). Similarly, a life history characterized by multiple familial disadvantages has been established as the defining feature of children described as “high risk” (35). While all family and household measures developed to date have been underpinned by assumptions, it is acknowledged that it is possible that these may not hold true in every circumstance.

In the family-weighted composite measure, for those indicators in which vulnerability increases with increasing prevalence, families were assigned the value 1 if one parent only displayed the characteristic and the value 2 if both parents, or a sole parent, displayed the characteristic. Binary variables were used for those indicators for which there is no corresponding increase in vulnerability with greater prevalence. For example, the presence of domestic violence within a home affects all family members equally (albeit in different ways). For these indicators, since *all* family members are affected, the value 2 was assigned if present. Finally, for longitudinally available variables, families were assigned the value 1 if the indicator was present in the past e.g., previous criminal offending, and the value 2 if the indicator was currently present. A total score of 3 was given to families who displayed persistent vulnerability on these indicators (e.g., long-term criminal offending). These weightings resulted in the family-weighted composite measure having a possible score range of 0 to 63. The method used for the weighting of indicators in the weighted composite measure of family-level vulnerability is presented in Table 1.

In assigning an overall vulnerability score in both the simple summation and the family-weighted composite measure, continuous scores were used in preference to categorical groupings (i.e., low, moderate and high vulnerability) to: (1) maximize the power for statistical analysis, (2) ameliorate subjectivity of defining cut points between groups, and (3) overcome the lack of population level data, which would usually be used to determine cut points.

Additionally, an exploratory factor analysis was performed to investigate whether differing “types” of vulnerability (based on grouping variables that were correlated) could be identified.

Correlation between simple summation and family-weighted composite scores and vulnerability types from the factor analysis was assessed. Whether the family-weighted composite score and the various vulnerability types differed by known demographic, antenatal, infant, and environmental risk factors for SUDI was then investigated.

### Statistical Analysis

All analyses were performed in SAS Version 9.4 (SAS Institute, Cary, NC, USA). Correlations and linear relationships between simple summation, family-weighted composite, and vulnerability type scores were assessed using proc reg. A strong relationship was found between the simple summation vulnerability score and the family-weighted composite score (*r*^2^ = 0.96). Due to its larger range and more detailed granularity the family-weighted composite score was used in all further analyses in relation to associations with known risk factors for SUDI.

Vulnerability types were determined using the initial binary variables created from the raw data identifying the known existence of the presence of each measure. Analysis was carried out using factor analysis with a varimax rotation (proc factor) such that the factors derived from the analysis were orthogonal to each other and thus independent. The number of factors was guided by the scree plot and assessment of intuitiveness of variables within factors, along with the number of variables comprising a factor. The weighting at which variables were considered substantially weighting on a factor was a balance of minimizing variables having a notable weighting to more than one factor, whilst limiting the number of variables not considered to be contributing to any factor. Variables were considered strongly associated with a factor when the factor loading was >0.40. The factors produced by this method are standardized and hence have a mean of 0 and a standard deviation of 1.

Associations between SUDI risk factors and both family-weighted vulnerability scores and vulnerability types were assessed using Wilcoxon tests for non-parametric data (Proc Npar1way). Differences in family-weighted vulnerability scores and vulnerability types were estimated using Hodges-Lehmann estimations with 95% confidence intervals. Sensitivity analyses were carried out using parametric statistics (*t*-tests, Proc *T*-test) and generalized linear models (Proc GLM). Effect sizes using parametric and non-parametric were found to be very similar.

## Results

A total of 228 cases of SUDI occurred in Queensland in the 5-year study period (2010–2014). Data on all social vulnerability indicators was complete, as if there was no information pertaining to an indicator, it was presumed that this vulnerability characteristic was not present. Data on SUDI risk factors was between 95 and 100% complete. For risk factor variables where data was not routinely collected (pillows, soft surfaces, and excess bedding), in this analysis, lack of mention of the presence of such a risk factor was presumed to mean that the factors was not present. Missing data for each risk factor variable is shown in Table 2.

**Table 2:** Association of family-weighted vulnerability score and vulnerability types by SUDI risk factors.

**SUDI risk factor**	**Family-weighted vulnerability score**	**Chaotic lifestyle**	**Socioeconomic**	**Psychosocial**
**DEMOGRAPHIC FACTORS**				
**Indigenous status**	***P*** **<** **0.0001**	***P*** **=** **0.003**	***P*** **<** **0.0001**	***P*** **=** **0.0004**
Non-Indigenous*	Ref	−0.12 (0.94)	−0.22 (0.93)	0.14 (0.93)
Indigenous	**6.2 (4.3, 8.1)**	**0.44 (0.13, 0.75)**	**0.84 (0.56, 1.12)**	**−0.52 (−0.83**, **−0.21)**
**Socioeconomic status**^**a**^ **(missing** **=** **1)**				
	***P=*** **0.002**	*P =* 0.17	***P=*** **0.0006**	*P =* 0.13
High*	Ref	−0.21 (0.13)	−0.42 (0.13)	0.18 (0.13)
Moderate	**3.6 (1.1, 6.0)**	0.21 (−0.18, 0.61)	**0.45 (0.07, 0.83)**	−0.30 (−0.61, 0.01)
Low	**3.4 (1.1, 5.6)**	0.30 (−0.01, 0.62)	**0.60 (0.30, 0.91)**	−0.07 (−0.47, 0.32)
**ANTENATAL AND BIRTH FACTORS**				
**Smoking during pregnancy (missing** **=** **7)**				
	***P<*** **0.0001**	***P=*** **0.0001**	***P<*** **0.0001**	*P =* 0.68
No*	Ref	−0.31 (0.85)	−0.30 (1.00)	−0.01 (0.61)
Yes	**5.5 (3.6, 7.3)**	**0.51 (0.26, 0.76)**	**0.54 (0.28, 0.80)**	0.06 (−0.21, 0.33)
**Small for gestational age (missing** **=** **3)**				
	*P =* 0.18	*P =* 0.11	*P =* 0.73	*P =* 0.48
>25th percentile*	Ref	−0.10 (0.10)	−0.04 (0.10)	−0.07 (0.10)
11–25th percentile	**2.6 (−0.3, 5.5)**	0.39 (0.03, 0.75)	0.15 (−0.22, 0.51)	0.21 (−0.15, 0.57)
<10th percentile	1.1 (−0.8, 3.0)	0.09 (−0.21, 0.38)	0.05 (−0.25, 0.34)	0.11 (−0.18, 0.41)
**Pre-term birth (missing** **=** **3)**				
	*P =* 0.24	*P =* 0.15	*P =* 0.11	*P =* 0.95
No*	Ref	0.04 (1.02)	−0.04 (0.99)	0.01 (1.02)
Yes	1.6 (−1.0, 4.1)	−0.28 (−0.66, 0.10)	0.31 (−0.067, 0.70)	0.01 (−0.37, 0.40)
**ENVIRONMENTAL FACTORS**				
**Breastfeeding (missing** **=** **6)**				
	***P=*** **0.01**	*P =* 0.93	*P =* 0.10	*P =* 0.09
Yes*	Ref	0.01 (0.96)	−0.14 (0.86)	−0.14 (0.83)
No	**3.7 (0.8, 6.7)**	0.01 (−0.25, 0.28)	0.22 (−0.03, 0.48)	0.22 (−0.02, 0.47)
**Sleep position (missing** **=** **12)**				
	***P=*** **0.001**	*P =* 0.09	***P=*** **0.04**	*P =* 0.18
Supine*	Ref	0.07 (0.08)	0.03 (0.08)	−0.01 (0.09)
Side	1.5 (−2.0, 4.9)	0.00 (−0.37, 0.37)	0.19 (−0.18, 0.56)	0.24 (−0.13, 0.61)
Prone	**−3.6 (−6.0**, **−1.4)**	**−0.37 (−0.72**, **−0.03)**	**−0.36 (−0.71, 0.02)**	−0.18 (−0.52, 0.17)
**Surface sharing (missing** **=** **0)**				
	***P=*** **0.0004**	***P=*** **0.0002**	***P=*** **0.04**	***P=*** **0.05**
No*	Ref	−0.21 (1.13)	−0.12 (1.00)	0.11 (1.00)
Yes	**3.5 (1.7, 5.3)**	**0.50 (0.23, 0.77)**	**0.28 (0.02, 0.54)**	**−0.26 (−0.52, 0.00)**
**Solitary sleep (excludes cases of surface sharing** **=** **97)**				
	***P<*** **0.0001**	***P=*** **0.0002**	***P=*** **0.0013**	*P =* 0.49
No*	Ref	0.18 (1.08)	0.16 (1.05)	−0.03 (0.99)
Yes	**−4.0 (– 6.0**, **−2.1)**	**−0.50 (−0.77**, **−0.24)**	**−0.44 (−0.71**, **−0.17)**	0.10 (−0.18, 0.37)
**Sleep on soft surface (missing** **=** **0)**				
	*P =* 0.29	*P =* 0.68	*P =* 0.38	*P =* 0.25
No*	Ref	−0.02 (0.99)	0.03 (1.030)	0.04 (1.06)
Yes	−1.0 (−2.8, 0.9)	0.07 (−024, 0.37)	−0.14 (−0.44, 0.17)	−0.18 (−0.48, 0.13)
**Pillows in environment (missing** **=** **0)**				
	*P =* 0.32	*P =* 0.80	*P =* 0.96	*P =* 0.40
No*	Ref	−0.01 (0.97)	−0.00 (1.06)	−0.05 (0.93)
Yes	0.9 (−0.9,2.7)	0.03 (−0.23, 0.30)	0.01 (−0.26, 0.27)	0.11 (−0.15, 0.38)
**Excess bedding (missing** **=** **0)**				
	***P=*** **0.01**	***P=*** **0.003**	*P =* 0.47	*P =* 0.96
No*	6.0 (0.5)	−0.16 (0.87)	−0.04 (0.99)	−0.00 (0.93)
Yes	**1.9 (0.5, 3.4)**	**0.40 (0.14, 0.66)**	0.10 (−0.17, 0.36)	0.01 (−0.26, 0.27)

* Reference group.

a *Socioeconomic status of area of residence, classified using the Socio-Economic Indexes for Areas (SEIFA)—Index of Relative Socio-Economic Advantage and Disadvantage. Bold is used to indicate statistical significance*.

### Distribution of Simple Summation and Family Weighted Composite Scores

Distribution of both simple summation and family-weighted composite scores were skewed and thus non-normally distributed. The simple summation score was distributed with a median of 5.7 (range 0–26), and the family-weighted composite score had a median of 7.5 (range 0–36.5). While highly correlated, a scatter plot demonstrated the ability of the family-weighted score to add a level of detail to that of the simple summation score, such that those subjects with any given score for the simple summation score showed variability on the family weighted composite score (Supplementary Figure 1).

### Associations Between Total Family Score and SUDI Risk Factors

Increased family vulnerability was associated with the presence of major known antenatal, birth, environmental and sociodemographic risk factors for SUDI (see Table 2). Significantly increased family vulnerability scores were observed among infants who were residing in low and moderate socioeconomic areas, exposed to tobacco smoke *in-utero*, born between the 11th and 25th percentile for weight, not breastfed at the time of death, sharing a sleep surface at the time of death, and exposed to excess bedding in the sleep environment. Conversely, two major environmental risk factors were observed among families of lower vulnerability; infants sleeping in a solitary sleep environment (i.e., separate sleep surface in separate room from an adult caregiver) and in the prone sleep position. There was a significant linear relationship between family vulnerability and the cumulative total of risk factors for SUDI (*P* = 0.002), family vulnerability increased with a greater number of risk factors (β = 1.17).

### Types of Social Vulnerability

Exploratory factor analysis using a varimax rotation, a three-factor solution and a weighting of 0.4 included 21 of the 32 indicators. These vulnerability types have been named chaotic lifestyle, socioeconomic, and psychosocial vulnerability. The indicators present in each vulnerability type (i.e., factor) are illustrated in Table 3. Variables that loaded onto the chaotic lifestyle vulnerability type included substance abuse (0.72), alcohol misuse (0.60), criminal offending (0.73) and long-term criminal offending (0.61), domestic violence (0.61), child abuse and neglect (0.63), children not in the care of biological parents (0.41), multiple partners (0.48), stressful life events (0.45), and transience and mobility (0.41). Variables that loaded onto the socioeconomic vulnerability type included low status parental occupations (0.70), unemployment (0.77), long-term unemployment (0.68), transience and mobility (0.45), lack of transport (0.53), limited access to primary care (0.40), and domestic violence in previous relationships. Variables that loaded onto the psychosocial vulnerability type were homelessness (0.43) and marginal homelessness (0.62), chronic illness or disability in parents (0.43), mental ill-health in parents (0.40), engagement in risky behaviors (sex work) (0.57), and domestic violence in previous relationships (0.43).

**Table 3:** Vulnerability types created through exploratory factor analysis of the simple summation scores.

**Social vulnerability indicators**	**Chaotic lifestyle**	**Socioeconomic**	**Psychosocial**
Material resources and financial hardship	0.34	0.37	0.27
Low status parental occupations	0.19	**0.70**	−0.17
Unemployment	0.14	**0.77**	−0.11
Long-term unemployment	0.20	**0.68**	0.11
Homelessness	0.04	0.38	**0.43**
Marginal homelessness	0.04	0.24	**0.62**
Overcrowding	0.26	0.17	−0.30
Transience and mobility	0.41	**0.45**	0.30
Chronic illness or disability in parents	0.26	0.01	**0.43**
Chronic illness or disability in siblings	0.05	0.27	0.12
Mental ill-health in parents	0.37	0.08	**0.40**
Alcohol misuse	**0.60**	0.33	0.05
Substance abuse	**0.72**	0.20	0.24
Liveborn sibling death	0.23	−0.06	−0.01
Previous stillbirth	0.15	−0.22	0.07
Multiple miscarriages or terminations	0.21	−0.16	0.36
Limited access to primary care (inc. antenatal)	0.21	**0.40**	0.00
Criminal offending	**0.73**	0.15	0.13
Long-term criminal offending	**0.61**	0.16	0.15
Victim of crime	0.37	0.35	−0.09
Domestic violence	**0.61**	0.20	−0.20
Domestic violence in previous relationships	0.14	**0.40**	0.43
Child abuse and neglect	**0.63**	0.37	0.17
Engagement in risky behaviors (sex work)	0.05	0.20	0.57
Lack of transport	0.01	**0.53**	0.35
Geographic isolation	0.07	0.14	−0.29
Limited social support	0.28	0.38	0.35
Multiple partners	**0.48**	0.15	0.33
Children not in care of biological parents	**0.41**	0.34	0.39
Stressful life events	**0.45**	0.30	0.32
Parental history of child abuse and neglect	0.20	0.37	0.19
Parental history of juvenile offending	0.12	0.27	0.07

In general, family-weighted composite vulnerability scores were not strongly correlated with any vulnerability type. There was a moderate correlation between family-type score and chaotic lifestyle (*r* = 0.68) and socioeconomic (*r* = 0.60) vulnerability types and a weak correlation with psychosocial vulnerability (*r* = 0.37). As the vulnerability types are independent because of the varimax rotation, a linear combination of the three scores accounts for effectively all on the variation when used to model the family weighted composite. This suggests that the vulnerability types identified through factor analysis are describing vulnerability in a more nuanced way than the total family-weighted composite score.

### Associations Between Vulnerability Type and SUDI Risk Factors

Chaotic lifestyle and socioeconomic vulnerability were associated with the presence of major known sociodemographic, antenatal and environmental risk factors for SUDI (see Table 2). Chaotic lifestyle vulnerability was significantly associated with smoking during pregnancy, surface sharing, and excess bedding. However, these families were less likely to place their infants prone to sleep and in a separate room from an adult caregiver (solitary sleep environments). Socioeconomic vulnerability was significantly associated with residing in both moderate and low socioeconomic areas, smoking during pregnancy and surface sharing. Again, these families were less likely to place their infants prone to sleep and in solitary sleep environments. Limited relationships between psychosocial vulnerability and SUDI risk factors were evident, with psychosocial vulnerability lower in those who were surface sharing.

There was a significant linear relationship between the total number of risk factors for SUDI and both chaotic lifestyle vulnerability (β = 0.09, *P* = 0.05) and socioeconomic vulnerability (β = 0.10, *P* = 0.02). There was no significant association between psychosocial vulnerability (β = 0.01, *P* = 0.89) and the number of SUDI risk factors.

### Social Vulnerability and Indigenous Identification

Infants who were identified as being Aboriginal and/or Torres Strait Islander had significantly higher family vulnerability scores than non-Indigenous infants (*P* = < 0.0001). Indigenous identification was also significantly associated with increased levels of chaotic lifestyle and socioeconomic vulnerability types. A positive association with psychosocial vulnerability was also observed, with the families of Indigenous infants having lower scores than non-Indigenous families to display this vulnerability type.

### Social Vulnerability and Cause of Death

There was no association between either family-weighted vulnerability scores or vulnerability types and cause of death classification.

## Discussion

Exploratory factor analysis identified three distinct types of social vulnerability, termed “chaotic lifestyle,” “socioeconomic,” and “psychosocial” vulnerability. Indicators in each vulnerability type were grouped together intuitively, based on what is already known about the co-occurrence of vulnerability characteristics. Studies have shown that lifestyle risk behaviors in the chaotic vulnerability type such as substance use, conflict, violence and abuse frequently co-occur resulting in chaotic home environments that have a deleterious effect on family functioning (36). Socioeconomic vulnerability largely corresponded with conventional indicators of socioeconomic disadvantage. The third vulnerability type, psychosocial vulnerability, involved mental health and homelessness for which the complex bidirectional relationship has been extensively described (37). Similarly, intimate partner violence (also included in the psychosocial vulnerability type) has been shown to be have detrimental effects on physical and psychological health, while at the same time poor psychological health is a risk factor for intimate partner violence (38).

As the level of social vulnerability experienced by families increased, there was a corresponding increase in the number of SUDI risk factors. Overall, families with higher levels of family-weighted vulnerability were more likely than families with less vulnerability to display four modifiable infant care practices: smoking during pregnancy, not breastfeeding, sharing a sleep surface, and the use of excess bedding. Two of the vulnerability types, chaotic lifestyle and socioeconomic vulnerability, were also positively related to the number of SUDI risk factors. All three vulnerability types were associated with surface sharing, and both chaotic lifestyle and socioeconomic vulnerability were also associated with maternal smoking during pregnancy. These are two major modifiable risk factors for SUDI, the combination of which has been shown to result in a 32-fold increased risk of infant death (39). Only chaotic lifestyle vulnerability was associated with the use of excess bedding. While there was a significant relationship between social vulnerability and not breastfeeding overall, artificial feeding was not significantly associated with any of the three vulnerability types.

While the socially patterned nature of both smoking and breastfeeding is established (40, 41), surface sharing has not been found to be more common among more socially deprived families (42). On the contrary, surface sharing is most prevalent among breastfeeding mothers (43). However, deaths in the context of surface sharing occur most frequently among Indigenous populations, racial and ethnic minorities, and other poor and marginalized people (44). There is growing consensus that it is not surface sharing itself but the circumstances in which it occurs, that places an infant at risk (43). The strong association between surface sharing and both chaotic lifestyle and socioeconomic vulnerability in this study suggests that a family's social environment may determine both the reason for shared sleep and the impact of surface sharing on the infant. Infant-parent surface sharing is frequently reported to be a socially and culturally valued part of infant care, influenced by parental philosophy, breastfeeding facilitation and convenience (45). However, shared sleep arrangements in the SUDI population may result less from ideology and personal choice than from the complex interplay of individual, social, and structural factors that shape the lives of more vulnerable families. This is in line with previous research that found that in some vulnerable populations shared sleeping may be used due to insufficient space for, or unavailability of a cot, or to keep infants safe (46). Importantly, no association was found between overall family-level social vulnerability or vulnerability types and cause of death. While it has been postulated that hazardous circumstances are suggestive that overlaying is a potential cause (43), and these results showed that hazardous circumstances increased with increasing vulnerability, this did not result in an increase in deaths due to suffocation among more socially vulnerable families. This indicates that the mechanisms through which social vulnerability exerts its effects on SUDI may not be as straightforward as presumed.

Although social vulnerability was generally associated with an increase in SUDI risk factors, families experiencing higher levels of vulnerability were less likely than families of lower vulnerability to place their infants prone to sleep, or in a separate room from an adult caregiver. While this is doubtless related in part to the higher incidence of surface sharing among more vulnerable families, the greater prevalence of prone and solitary sleep among families with lower levels of vulnerability is consistent with the findings of a recent Queensland infant care practice study with a socially advantaged sample (47). Cole et al. (47) reported that 17% of infants were placed non-supine to sleep (prone or side) and 25% infants slept in a room alone. Previous epidemiological studies have demonstrated that being placed prone for sleep is a causal risk factor for sudden infant death (1). Studies also suggest that infants who sleep in a separate room from an adult caregiver are at an increased risk of sudden death, with room-sharing estimated to reduce the risk of SUDI by up to 50% (48).

Indigenous infants were found to experience higher levels of social vulnerability than other families. Indigenous identification was also associated with chaotic lifestyle and socioeconomic vulnerability. Indigenous infants were less likely to experience psychosocial vulnerability than non-Indigenous infants, although the reasons for this are unclear. Recent research has also demonstrated that number of antenatal, birth, environmental and sociodemographic risk factors for SUDI, which are also related to the social determinants of health, have a higher prevalence among Indigenous compared with non-Indigenous infants (23). With a SUDI rate over three times that of non-Indigenous infants (25), the significantly higher levels of social vulnerability experienced by Indigenous families are likely to at least in part account for the previously reported disparity between Indigenous and non-Indigenous SUDI. It is also likely to reflect likely reflect the ongoing disadvantage, persisting over time and across generations, that continues to be experienced by Aboriginal and Torres Strait Islander Australians, which occurs as a result of colonization and dispossession, structural racism, and the lack of culturally competent services (49).

The relationship between area level socioeconomic status and social vulnerability in this study is also noteworthy. While families who lived in lower socioeconomic areas of Queensland generally experienced higher levels of social vulnerability than those residing in more affluent areas, area-level disadvantage was only associated with socioeconomic vulnerability. This suggests that previous research using only single or a limited set of indicators of economic disadvantage, such as deprivation of area of residence (2), parents' occupation (50), or level of education (51), may have neither captured all facets of social vulnerably nor dealt effectively with the complexity of multiple disadvantage.

The findings of this study have a number of implications. Over the past two decades SUDI rate reductions have slowed and plateaued. If SUDI rates are to be further reduced, the way in which risk reduction strategies are prioritized and distributed needs to be re-evaluated. These results indicate that lower and higher vulnerability families display different risk factors for SUDI, and that it is therefore unlikely that a single preventative approach will achieve universal success in reducing SUDI mortality in Australia. Proportionate universalism is an approach that balances targeted and universal public health perspectives through action proportionate to needs and levels of social vulnerability in a population (52).

Previously, health promotion campaigns that revolved around one, simple key message advising parents to place infants on their backs to sleep, achieved notable infant mortality reductions in middleclass, white communities (53). As other risk factors, most notably smoking, have overtaken non-supine sleep in terms of both prevalence and importance, most countries have progressively moved away from “Back to Sleep” as a singular message. The current Australian Safe Sleeping campaign contains six messages, which have low adherence (<13%) by socially advantaged families in Queensland (47). Providing families with a list of recommendations may create an inherent complexity which encourages families to pick and choose only the ones that appear to suit their needs (54). “Developing and evaluating new ways to make safe sleeping campaigns more effective” has been identified as a key Australian SUDI research priority (55). This research indicates promotion of the back-sleeping position and the need for close infant-parent proximity during sleep continues to be of paramount importance to any mainstream SUDI reduction campaign.

This study identifies families experiencing multiple, co-occurring adversities as a priority population. The multiple disadvantage experienced by these families means that individually tailored or community-based, complex, multimodal interventions, such as wrap around care approaches, may be needed if reductions in SUDI mortality are to be achieved among socially vulnerable groups. In order to be successful, interventions need to be relevant to, meet the needs of, and recognize the competing priorities faced by the individuals and groups targeted (56). This means that instead of focusing solely on the elimination of risk behaviors, they must recognize the broader social, cultural, and structural factors at play, which influence the way individuals functioning in complex situations make decisions about infant care (7).

### Limitations

It would be usual to test a social vulnerability framework in the general population before applying it to a specific, narrower population. Unfortunately, this is beyond the scope of the present study, which was a retrospective cohort study and therefore did not have the advantage of control data. Consequently, the population prevalence of social vulnerability among families with infants and young children could not be estimated. To overcome this limitation in future studies, the Normative SVF is currently being piloted at population level, by mapping measures identified in the Normative SVF against variables in the administrative datasets available in the New Zealand Integrated Data Infrastructure (IDI)^1^. The IDI contains person level linked longitudinal microdata about individuals from New Zealand government agencies (including but not limited to, taxable income, hospitalizations and other contacts with the health system, education status, criminal convictions, social welfare benefits received, involvement with child protection and youth justice systems) (57). This pilot may involve data reduction to produce a shortened version of the instrument, including the removal of indicators that have little variance or were found to result in large amounts of missing data. There may be substantial potential for linked data to standardize collection of variables relevant for measuring social vulnerability. The study dataset comprises routinely collected administrative data that does not have perfect correspondence with what is necessarily most relevant for measuring social vulnerability and some important elements, such as income and education, were unable to be included. Additionally, since data on indicators were predominantly extracted from narrative text, where there was no information pertaining to an indicator, it was presumed that this vulnerability characteristic was not present. In these circumstances there is an implicit lack of disadvantage assigned to families. Consequently, it is likely that the results may be conservative and that the level of social vulnerability among families who have experienced a SUDI has been underestimated. This may in part explain the unexpected finding that psychosocial vulnerability was not related to either the cumulative total of risk factors or any specific risk factor, and the finding that psychosocial vulnerability was lower in those who were surface sharing.

### Strengths

The present study is one of only a limited number to attempt to estimate family-level social vulnerability using a multidimensional approach, which incorporates dimensions beyond the purely economic. Perhaps more importantly, it is the first to apply a multidimensional measure to investigating associations between social vulnerability level and types and risk factors for sudden infant death. In so doing, this study provides the first response to respond to calls to use sociological paradigms to develop a multifactorial measure of disadvantage for use within SUDI research (4, 7). It also responds to the recent recommendation that future studies assess SUDI risk factor prevalence differences among population subgroups (47). Although applied in the Australian context, it is likely that the Normative SVF will have broad applicability in Western countries with similar social, political and economic systems, where similar epidemiological changes in SUDI have been observed, including the United Kingdom, the United States, Canada, and New Zealand. The critical role of social factors in children's health has long been recognized, and the importance of understanding the multifactorial nature of disadvantage is well-established (58). The Normative SVF may also be a useful tool to assess potential relationships between multiple adversity and other potentially preventable mortality and morbidity in pregnancy, infancy, and early childhood. This will be a focus of future research.

## Conclusion

Social vulnerability has many manifestations extending well-beyond economic deprivation and can only be adequately measured using a multidimensional model that captures disadvantage across a wide range of indicators. Study results have highlighted the continued importance of promoting back-sleeping in mainstream information sharing public health campaigns. However, understanding the combined effect of social vulnerability and SUDI risk is of paramount importance for policy making and resource allocation in the context of primary prevention. This study demonstrates that socially vulnerable families are at increased risk of sudden infant death due to the concentration of SUDI risk factors in these families and should be a priority group for tailored action.

## Data Availability Statement

The data analyzed in this study is subject to the following licenses/restrictions: The datasets used and/or analyzed during the current study are not available publicly as this was not specified in the original ethical approval request, however, may be available in part from the corresponding author on reasonable request. Requests to access these datasets should be directed to Rebecca Shipstone, rebecca.shipstone@research.usc.edu.au.

## Ethics Statement

The studies involving human participants were reviewed and approved by University of the Sunshine Coast Human Research Ethics Committee (HREC: S/15/805). Due to the retrospective nature of the study, ethical approval included a waiver of the requirement for informed consent from the participants' legal guardian/next of kin to participate in this study.

## Author Contributions

RS conceptualized the study, undertook literature searches, collected data, contributed to data analysis and interpretation, and led the manuscript preparation (including drafting the original manuscript). JT performed data analysis, interpreted, analyzed data, assisted with manuscript preparation, critically reviewed and approved the final manuscript for submission, and provided study supervision. JY and LK assisted with study conceptualization, provided study supervision and intellectual input, and critically reviewed and approved the final manuscript for submission. All authors contributed to the article and approved the submitted version.

## Conflict of Interest

The authors declare that the research was conducted in the absence of any commercial or financial relationships that could be construed as a potential conflict of interest.

## Footnotes

^1^Australia does not have national or state-based data sets containing routinely linked administrative records of individuals' contacts with government agencies, accessible for research purposes. As such piloting the SVF in Australia would have involved a large scale, costly data linkage project. Given the historical, social, political, and economic similarities between the two countries, and the accessibility of linked microdata, New Zealand was considered an ideal testing ground for the piloting of the SVF at population level.
