# Supplementary material for: Applying a Social Exclusion Framework to Explore the Relationship Between Sudden Unexpected Deaths in Infancy (SUDI) and Social Vulnerability
Source: Front Public Health. 2020 Oct 20;8:563573. doi: 10.3389/fpubh.2020.563573 (PMC7606531; doi:10.3389/fpubh.2020.563573)
Supplement: Supplementary file 1 [file Table_1.DOCX]

**Supplementary Table 1: Comparison of major social exclusion frameworks and indicators**

| Indicators | | *Laeken^1^* | *Atkinson^2^* | *CASE^3^* | *Opportunity for All^4^* | *New Policy Institute^5^* | *PSE Survey^6^* | *B-SEM^7^* | *CUPSE^8^* | *Scutella*  *et al.^9^* | *AISB^10*^* | *Child SE^11^* |
| --- | --- | --- | --- | --- | --- | --- | --- | --- | --- | --- | --- | --- |
|  |  | EU | EU | UK | UK | UK | UK | UK | AUS | AUS | AUS | AUS |
| *Material & economic resources* | Low income (under 60% or 50% or other) | • | • | ❖ | • | • | ❖ | ❖ |  | ❖ | • | • |
|  | Persistence of low income | • |  | ❖ |  | • |  |  |  |  |  |  |
|  | Low wealth (low net worth) |  |  |  |  |  |  |  |  |  |  |  |
|  | Welfare reliance |  |  |  | • | • |  |  |  | ❖ | • |  |
|  | Material deprivation (can’t afford necessities) |  |  |  |  |  | ❖ | ❖ | ❖ |  | • |  |
|  | Financial hardship (can’t pay pills, in debt) |  | • |  |  |  |  |  | ❖ |  |  |  |
|  | Without bank account or access to bank |  |  |  |  | • |  | ❖ | ❖ |  |  |  |
|  | Subjective poverty |  |  |  |  |  | ❖ | ❖ | ❖ |  |  |  |
| *Employment* | Underemployment (forced part-time, job insecurity) |  | • |  |  | • |  | ❖ |  |  |  |  |
|  | Unemployment |  | • | ❖ | • | • | ❖ | ❖ | ❖ | ❖ | • |  |
|  | Long term unemployment | • | • |  |  |  |  |  |  | ❖ |  |  |
|  | Jobless households | • | • |  | • | • | ❖ |  | ❖ |  | • | • |
|  | Occupation type (semi/unskilled/low paid) |  | • |  |  | • |  | ❖ |  |  |  | • |
| *Education & skills* | School attendance and/or exclusions |  |  |  | • | • |  | ❖ |  |  |  |  |
|  | Limited educational attainment | • | • |  | • | • |  | ❖ |  | ❖ | • | • |
|  | No advanced or vocational education | • | • |  |  | • |  |  |  |  | • |  |
|  | Low literacy/numeracy |  | • |  | • |  |  | ❖ |  | ❖ | • |  |
|  | Low communication (incl. English skills) |  | • |  |  |  |  | ❖ | ❖ | ❖ |  | • |
| *Health* | Morbidity, mortality and life expectancy | • | • |  | • | • |  |  |  |  | • |  |
|  | Disability |  |  |  | • | • | ❖ |  |  | ❖ | • |  |
|  | Limiting longstanding illness |  |  |  |  | • |  | ❖ |  |  | • |  |
|  | Mental illness |  |  |  |  | • |  | ❖ |  | ❖ |  |  |
|  | Lack of access to health/disability services |  | • |  |  |  |  |  | ❖ |  | • |  |
|  | Dental health |  |  |  |  | • |  |  |  |  |  |  |
|  | Poor self-defined health status | • | • |  |  |  |  | ❖ |  | ❖ | • |  |
|  | Use of alcohol and/or substance abuse |  |  |  | • | • |  | ❖ |  |  |  |  |
|  | Other health issues (low birthweight, obesity smoking, teen pregnancy, suicide rates) |  |  |  | • | • |  |  |  |  | • |  |
| *Housing* | Housing tenure (incl. homelessness, unmet need) |  | • |  | • | • |  |  |  |  | • | • |
|  | Low housing quality/lacking amenities |  | • |  | • | • |  |  |  |  |  |  |
|  | Utility disconnections |  |  |  |  |  |  | ❖ |  |  |  |  |
|  | Overcrowded housing |  |  |  |  | • |  |  |  |  |  |  |
| *Crime & safety* | Criminal record, incarceration, antisocial behaviour |  |  |  |  |  |  | ❖ |  | ❖ |  |  |
|  | Victim of crime, threats to personal safety |  |  |  | • | • |  | ❖ |  | ❖ | • |  |
|  | Unsafe neighbourhood/fear of crime |  |  |  | • | • | ❖ | ❖ |  | ❖ | • |  |
|  | Child abuse & neglect |  |  |  | • |  |  |  |  |  | • |  |
|  | Living in a disadvantaged area |  |  |  | • |  |  |  |  |  |  |  |
| *Social participation* | Single parent/absence of a parent |  |  |  | • |  |  |  |  |  |  | • |
|  | Lacks family and social support |  |  | ❖ |  |  | ❖ | ❖ | ❖ | ❖ | • |  |
|  | Lacks regular social contact |  |  | ❖ |  |  | ❖ | ❖ | ❖ |  | • |  |
|  | Low social/community/political participation |  | • |  |  | • | ❖ | ❖ | ❖ | ❖ | • |  |
|  | Access to public and private services |  | • |  |  |  | ❖ | ❖ |  | ❖ | • |  |
|  | Minority groups and discrimination |  |  |  |  |  |  | ❖ |  |  |  |  |
| *Mobility* | Lack of transport (incl. unable to afford fuel) |  |  |  | • | • |  | ❖ | ❖ | ❖ |  | • |
| - Indicators collected at individual level   ● Indicators collected at population level  *The AISB used a combination of individual level (collected through national surveys) and population level data. Where possible this is indicated.  1. Lakean Indicators – the European Union’s core set of poverty and social exclusion indicators (1)  2. Atkinson Indicators – a more expansive set of indicators proprosed by the EU Social Protection Committee (2)  3. Centre for the Analysis of Social Exclusion (CASE) (UK) (3)  4. Department of Social Security (UK) ‘Opportunity for All’ audit of poverty and social exclusion (4).  5. New Policy Institute (UK) (5)  6. Millennium Survey of Poverty and Social Exclusion (PSE) (UK) – first nationally representative survey measuring social exclusion (6)  7. Bristol Social Exclusion Matrix (B-SEM) – most extensive suite of indicators on social exclusion and vulnerability (7)  8. Social Policy and Research Centre ‘Community Understanding of Poverty and Social Exclusion’ (CUPSE) survey (8)  9. Melbourne institute of Applied Economic and Social Research (9)  10. Australian Government’s Social Inclusion Unit (10)  11. Child Social Exclusion Index (Australia) (11) | | | | | | | | | | | | |

**References**

1. Social Protection Committee (EU). Report on indicators in the field of poverty and social exclusion. Brussells: Council of the European Union, (2001).

2. Atkinson T, Cantillon B, Marlier E, Nolan B. *Social indicators: the EU and social inclusion*. Oxford: Oxford University Press (2002).

3. Burchardt T, Le Grand J, Piachuad D. Degrees of exclusion: developing a dynamic, multidimensional measure. In: Hills J, Le Grand J, Piachuad D, editors. *Understanding social exclusion*. Oxford: Oxford University Press (2002). p. 30-43.

4. Department of Social Security. *Opportunity for all: tackling poverty and social exclusion.* London: Department of Social Security (1999).

5. Palmer G, Joseph Rowntree Foundation. *The poverty site: the UK site for statistics on poverty and social exclusion United Kingdom.* London: Joseph Rowntree Foundation (2002) [cited 15 March 2019]. Available from: http://www.poverty.org.uk/​

6. Levitas R. The concept and measurement of social exclusion. In: Pantazis C, Gordon D, Levitas R, editors. *Poverty and social exclusion in Britain*. Bristol: The Policy Press (2006). p. 123-160.

7. Levitas R, Pantazis C, Fahmy E, Gordon D, Lloyd E, Patsios D. T*he multi-dimensional analysis of social exclusion.* Bristol: University of Bristol (2007).

8. Saunders P, Naidoo Y, Griffiths M*. Towards new indicators of disadvantage: deprivation and social exclusion in Australia.* Sydney: Social Policy Research Centre (2007).

9. Scutella R, Wilkins R, Horn M. *Measuring poverty and social exclusion in Australia: a proposed mulidimensional framework for identifying socio-economic disadvantage*. Melbourne: Melbourne Institute of Applied Economic and Social Research, University of Melboune (2009).

10. Australian Social Inclusion Board (ASIB). *Social inclusion in Australia: how Australia is faring, 2nd Edition*. Canberra: Department of the Prime Minister and Cabinet (2012).

11. McNamara J, Harding A, Daly A, Tanton R. *Child social exclusion: an updated index from the 2006 Census*. Canberra: National Centre for Social and Economic Modeling (NATSEM), University of Canberra, (2008).
