# Supplementary material for: Applying a Social Exclusion Framework to Explore the Relationship Between Sudden Unexpected Deaths in Infancy (SUDI) and Social Vulnerability
Source: Front Public Health. 2020 Oct 20;8:563573. doi: 10.3389/fpubh.2020.563573 (PMC7606531; doi:10.3389/fpubh.2020.563573)
Supplement: Supplementary file 2 [file Table_2.DOCX]

**Supplementary Table 2: Domains and Indicators of Social Vulnerability – Conceptual Justification**

| Income and material resources |
| --- |
| The ‘income and material resources’ domain encompasses conventional concepts of poverty and deprivation. Numerous studies demonstrate that children who live in poor households are more likely to experience long-term, suboptimal outcomes, including psychological consequences, which leave people ill-equipped to improve their life circumstances (1, 2). This domain includes one income-based measure; family income below the poverty line, measured by receipt of a social care program for people with limited resources. In the Australian context, this is most readily measured by holding a health-care card (an Australian government concession scheme providing people with low income access to reduced cost medical expenses and other concessions). Holding a health-care card has been used in recent Australian research as a proxy for low income (3). Low income is conventionally measured at 50% or 60% of the median income. The latest Australian figures show that 50% of median income equates to a disposable income of less than $895 per week, for a couple with two dependent children (4). By comparison, a working couple with two dependent children must have an income below $1007 per week to be eligible for a low-income Health Care Card.  The use of this proxy indicator is preferred to an explicit income measure, because requesting information about access to a concessional benefits scheme is less invasive and may be available through data linkage. Material deprivation and hardship measures compliment income based measures by directly assessing the impact of lack of income and resources (5, 6). The indicators of financial hardship are an abridged version of the hardship items used in Australian longitudinal population-based surveys (7, 8), and are a standard measure of basic lifestyle deprivation used in numerous studies (5, 6, 9). Indicators of limited material resources and financial hardship included in this domain are: inability to afford adequate food and/or clothing (including infant specific items), inability pay rent or mortgage on time, and pawning or selling possessions to buy necessities or seeking assistance from welfare organisations, over the past 12 months. |
| **Employment** |
| A lack of participation in the labour market is central to social exclusion, and thus joblessness is a key indicator of social exclusion. Precarious positions (those with little job security) are associated with a ‘low pay no pay’ cycle. People employed in these positions are likely to face unemployment within a year, and when re-employed, are more likely to be lowly paid (10). Welfare dependence also has psycho-social effects beyond economic ramifications (1, 11). While some researchers subsume employment within the domain of income and material resources, following both Scutella and colleagues (12, 13) and Levitas and colleagues (14), this has been treated as a separate category because it has importance to social vulnerability independent of the financial effects. Indicators in this domain are under and precarious employment, welfare reliance, unemployment, and long-term unemployment. |
| **Education and skills** |
| Individuals with low levels of educational attainment and poor basic skills are at a high risk of social exclusion (15, 16). Educational attainment has a well-known impact on employment and occupation, and thus financial security. There is also a documented association between parental education and child development and outcomes (17). Low attainment is correlated with poor mental and physical health, and lower levels of civic participation (10). Poor literacy and numeracy have also been found to be of equal importance in social exclusion (15). Indicators included in this domain are early and very early school leavers, having no vocational education or training, and poor basic skills (literacy, numeracy, competence in English). |
| Housing and living environment |
| Decent quality and stable housing are some of the most essential resources for long term wellbeing. Homelessness is the most extreme form of social disadvantage in housing (10). It includes situations in which people are sleeping rough, couch surfing, or staying in homeless shelters or hostels. However, homelessness also includes a variety of precarious, insecure forms of accommodation, such as boarding houses and caravans, and make-shift accommodation such as sheds, tents, or converted garages. This latter form of homelessness has been described as ‘hidden homelessness’ (18) and ‘living on the margins of the housing market’ (10). Studies in Australia (19) and Canada (20) have shown that the problems accompanying homelessness may be further exacerbated in rural and remote areas. The combination of vast, sparsely inhabited localities, a harsh climate, and limited access to required services provides unique challenges for those without adequate housing in these areas (20). Studies have recently raised concern about dwellings that provide inadequate shelter from the elements, are unfit for human habitation, and/or are extremely overcrowded (21). A lack of basic services inside the home such as gas, electricity water, or a telephone, can also be seen as housing deprivation and exclusion from the most basic of services (22). Indicators in this domain are transience and homelessness, public housing, poor housing quality, lack of basic housing facilities, and overcrowding |
| **Health and healthcare** |
| Health status and access to healthcare are measured as a consistent part of social exclusion in more comprehensive frameworks (12-14, 22, 23). At least five subdomains of relevance have been identified in the research literature as health drivers of social exclusion: chronic or longstanding conditions, disability, alcohol and other substance misuse, poor mental health, and accidental child deaths. There are clear social gradients in the areas of development, health, and wellbeing among Australian children (24), with injury being the health event in children with the steepest social gradient, and the closest relationship with deprivation and social exclusion (10). The nexus between social exclusion and disability has been examined in a number of studies (25-28). Delays and impairments may be caused by social exclusion (mediated via exposure to environmental adversity and psychosocial hazards) (26). At the same time, families supporting a member with disabilities or chronic health issues are at increased risk of social exclusion themselves due in part to the financial impact of providing this support, as well as their more limited opportunities for social and cultural engagement (13, 28). Alcohol and other substance misuse also have a deleterious impact on family functioning. Symptoms of intoxication or withdrawal impact on a parent’s ability to maintain household tasks, roles, and responsibilities necessary for the wellbeing of children, and may result in inconsistent parenting (29, 30).  There is a growing body of research investigating access to health services and social exclusion. A number of these studies use ambulatory care-sensitive conditions (ACSCs) as a marker for access to timely and effective primary care (31-33). Also known as potentially preventable hospitalisations, ACSCs are considered avoidable with the application of public health interventions and early disease management, delivered in a primary care setting. In children, ACSCs include conditions such as asthma, bacterial pneumonia, dehydration, vaccine preventable diseases, and failure to thrive. In addition, untreated or recurrent parasitic infestations such as scabies and/or head lice in children are a well-documented indicator of social deprivation, neglect, and overcrowded living conditions (34). Inadequate attendance at antenatal care (i.e. late initiation and/or fewer visits) among marginalised women has also been found to be linked to a number of interrelated personal, structural and health provider barriers (35). These factors are considered to provide indirect evidence of problems with patient access to or use of primary healthcare (including less active care seeking and poorer health literacy), inadequate skills and resources, or disconnection with specialist services (32). Indicators this domain are chronic illness, condition or disability, mental ill-health, self-harm and suicide, substance misuse and drug diversion therapy, avoidable infant and child mortality, lack of access to health services and less than adequate antenatal care. |
| Crime and threats to personal safety |
| Crime and threats to personal safety can impact on an individual’s participation in a wide range of economic, social, and civic activities, and are well established as both indicators and drivers of social exclusion (13). Research has established that social exclusion affects not only the victims of crime, but also the perpetrators. For example, a criminal record may lead to exclusion by having an impact on future employment chances (10, 36). Family violence, and sexual and physical assault both push people into social exclusion, and worsen the experience of exclusion for those most vulnerable (24). The majority of families known to child protection services are also socially excluded, with the indicators of social exclusion mirroring common risk factors for maltreatment (29). Children known to child protection are an extremely vulnerable population having suffered harm and/or being at risk of future harm (24).  Women involved in all forms of sex work (and in particular, those involved in street prostitution and prostitution during pregnancy) face perpetual risks to their personal safety, in the form of physical and sexual violence, as well as health risks such as sexually transmitted infections (37, 38). These health risks endanger women’s lives and wellbeing and, if pregnant, pose serious threats to a fetus and subsequent infant. Studies have shown women involved in prostitution during pregnancy experienced very poor reproductive outcomes, with less than 60% of pregnancies resulting in the live birth of an infant who survived past their first birthday (37). Many of the factors that predispose women to engage in sex work are both indicators and drivers of social exclusion. Indicators in this domain are recent victim of crime, recent perpetrator of crime, long term criminal offending history, domestic violence, engagement in risky behaviours (including all forms of sex work), and child abuse and neglect. |
| **Transport and access to services** |
| The link between social exclusion and transport has been increasingly discussed in the literature (39, 40). A lack of adequate transport contributes to social exclusion by restricting involvement in the economic, political, and social life of a community. According to Kenyon, Lyons (41), exclusion in transport occurs either in whole or in part due to insufficient mobility in a society and environment built around the assumption of high mobility. Cars have become increasingly necessary to reach work, shops, health services, and schools, especially for families, and income poor people without access to a car make far fewer, shorter, and slower journeys (10, 40). Families with infants and young children, or with disabled members, experience problems accessing public transport. For those reliant on public transport, accessing health services is notoriously difficult, and frequently leads to missed health appointments. Australian researchers have identified that acute cases of transport exclusion are predominantly found in suburban and regional areas, where distance is a major barrier to inclusion (42, 43). Indicators in this domain are carless households, difficulty accessing services due to limited or inaccessible public transport or periodic inability to re-fuel and/or run a car, and geographic isolation (defined as those areas where geographic distance imposes a high or very high restriction upon accessibility to goods, services, and opportunities for interaction). |
| **Family and social relationships** |
| This domain refers to interaction with, and support from, families and friends, and engagement in society more generally. It has previously been observed that the indicators in this domain are more difficult to collect and measure objectively than in other domains (13). The social environment is acknowledged as a multifaceted determinant of health (44). The contribution of social embeddedness to social inclusion has been widely recognised. The two main theories of social embeddedness focus on reciprocal interpersonal relationships (social support) (45), and on broader neighbourhood networks, the amount and quality of which are integrally linked to the areas in which individuals live (social capital) (46). Positive social networks, have been shown to function as buffers, contributing to family resilience and ameliorating the effects of social exclusion on health by increasing the capacity to withstand life stressors (22, 47, 48). Conversely, limited social ties and/or noxious relationships are associated with a host of undesirable outcomes, including increased morbidity and mortality (49). There are a number of standardised, validated tools for measuring social support (48, 50) and stressful life events (51), which have been used extensively in studies of health, including SUDI (52). Indicators in this domain are limited social support, families with children born to multiple partners, families with children not in the care of biological (or adoptive) parents, and stressful life events. |
| **Intergenerational transfer of disadvantage** |
| Until relatively recently the intergenerational transfer of disadvantage, the situation in which more than one generation of the same family experience high levels of disadvantage, has received only scant attention within the literature (24). Substantial research shows that childhood experience of trauma and adversity contributes to social disadvantage and exclusion in adulthood (29, 53, 54). Parental unemployment, substance misuse, homelessness, domestic violence, and physical and mental health problems are frequently associated with past histories of child abuse and neglect and involvement in the juvenile justice system (54-57). Indicators in this domain are parental history of child abuse and/or neglect as children and parental history of juvenile offending. |

# References

1. Daly A, Smith D. Indicators of risk to the wellbeing of Australian Indigenous children. *Australian Review of Public Affairs* (2005) 6(1):39-57.

2. Gordon D, Levitas R, Pantazis C, Patsios D, Payne S, Townsend P, et al. *Poverty and social exclusion in Britain*. York: Joseph Rowntree Foundation (2000).

3. Terry D, Ervin K, Soutter E, Spiller R, Nogare ND, Hamilton AJ. Do not "let them eat cake": correlation of food-consumption patters among rural primary school children from welfare and non-welfare households. *Int J Env Res Public Health* (2017) 14(1):26. doi: 10.3390/ijerph14010026.

4. Australian Council of Social Service. *Poverty in Australia 2016.* Strawberry Hills: Australian Council of Social Service (2016) [cited 2020 January 24]. Available from: http://www.acoss.org.au/wp-content/uploads/2016/10/Poverty-in-Australia-2016.pdf.

5. Butterworth P, Crosier T. Deriving a measure of fi nancial hardship from the HILDA survey. *Australian Social Policy* (2005):1-12.

6. Nolan B, Whelan CT. Using non-monetary deprivation indicators to analyze poverty and social exclusion: lessons from Europe? *J Policy Anal Manage* (2010) 29(2):305-25. doi: 10.1002/pam.20493.

7. Australian Institute of Family Studies. *The longitudinal study of Australian chidren: Annual statistical report 2014.* Melbourne: Australian Institute of Family Studies (2015) [cited 2020 January 24]. Available from: https://aifs.gov.au/publications/longitudinal-study-australian-children-annual-statistical-report-2014.

8. Marks GN. *Income proverty, subjective poverty and finiancial stress. Social policy research paper No. 29*. Canberra: Department of Families, Community Services and Indigenous Affairs (2007).

9. Nolan B, Whelan CT. *Resources, deprivation and poverty*. Oxford: Oxford University Press (1996).

10. Bradshaw J, Kemp P, Baldwin S, Rowe A. *The drivers of social exclusion: a review of the literature for the Social Exclusion Unit in the Breaking the Cycle series*. London: Social Exclusion Unit, Office of the Deputy Prime Minister (2004).

11. Reid C, Herbert C. ‘Welfare moms and welfare bums’: revisiting poverty as a social determinant of health. *Health Sociol Rev* (2005) 14(2):161-73. doi: 10.5172/hesr.14.2.161.

12. Scutella R, Wilkins R, Kostenko W. Intensity and persistence of individuals' social exclusion in Australia. *Australian Journal of Social Issues* (2013) 48(3):273-98.

13. Scutella R, Wilkins R, Horn M. *Measuring poverty and social exclusion in Australia: a proposed mulidimensional framework for identifying socio-economic disadvantage.* Melbourne: Melbourne Institute of Applied Economic and Social Research, University of Melboune (2009).

14. Levitas R, Pantazis C, Fahmy E, Gordon D, Lloyd E, Patsios D. *The multi-dimensional analysis of social exclusion*. Bristol: University of Bristol (2007).

15. Bynner J, Parsons S. Social exclusion and the transition from school to work: the case of young people not in education, employment, or training (NEET). *J Vocat Behav* (2002) 60(2):289-309. doi: 10.1006/jvbe.2001.1868.

16. Hick P, Visser J, McNab N. Education and social exclusion. In: Abrams D, Christian J, Gordon D, editors. *Multidisciplinary handbook of social exclusion research*. West Sussex: John Wiley & Sons (2007). p. 95-114.

17. Sparkes J, Glennerster H. Preventing social exclusion: education's contribution. In: Le Grand J, Piachuad D, editors. *Understanding social exclusion*. Oxford: Oxford University Press (2002). p. 178.

18. Reeve K, Batty E. *The hidden truth about homelessness: expereince of single homelessness in England*. London: Crisis (2011).

19. Grigg M, Judd F, Ryan L, Komiti A. Identifying marginal housing for people with a mental illness living in rural and regional areas. *Australas Psychiatry* (2004) 12(1):36-41. doi: 10.1046/j.1039-8562.2003.02057.x. PubMed PMID: 15715737.

20. Waegemakers Schiff J, Schiff R, Turner A. Rural homelessness in western Canada: lessons learned from diverse communities. *J Soc Incl* (2016) 4(4):73-85. doi: 10.17645/si.v4i4.633.

21. Bramley G, Besemer K. *Housing and the living environment indicators in the PSE survey, working paper, methods series No. 6.* United Kingdom: Economic and Social Research Council (2011) [cited 2020 May 2, 2020]. Available from: https://www.poverty.ac.uk/system/files/attachments/WP%20Methods%20No.6%20-%20Housing%20Living%20Environment%20Indicators%20(Besemer,%20Bramley).pdf.

22. Levitas R. The concept and measurement of social exclusion. In: Pantazis C, Gordon D, Levitas R, editors. *Poverty and social exclusion in Britain*. Bristol: The Policy Press (2006). p.123-160.

23. Palmer G, Joseph Rowntree Foundation. *The poverty site: the UK site for statistics on poverty and social exclusion.* United Kingdom: Joseph Rowntree Foundation (2002) [cited 15 March 2020]. Available from: http://www.poverty.org.uk/​

24. Hayes A, Gray M, Edwards B*. Social inclusion: origins, concepts and key themes.* Canberra: Social Inclusion Unit, Department of Prime Minister and Cabinet (2008).

25. Emerson E. Poverty and people with intellectual disabilities. *Ment Retard Dev Disabil Res Rev* (2007) 13(2):107-13. doi: 10.1002/mrdd.20144.

26. Emerson E. Commentary: childhood exposure to environmental adversity and the well-being of people with intellectual disabilities. *J Intellect Disabil Res* (2013) 57(7):589-600. doi: 10.1111/j.1365-2788.2012.01577.x.

27. Leonard H, Petterson B, De Klerk N, Zubrick SR, Glasson E, Sanders R, et al. Association of sociodemographic characteristics of children with intellectual disability in Western Australia. *Soc Sci Med* (2005) 60(7):1499-513. doi: 10.1016/j.socscimed.2004.08.014.

28. Gilroy J, Emerson E. Australian indigenous children with low cognitive ability: family and cultural participation. *Res Dev Disabil* (2016) 56:117-27. doi: 10.1016/j.ridd.2016.05.011.

29. Bromfield L, Lamont A, Parker R, Horsfall B. *Issues for the safety and wellbeing of children in families with muliple and complex problems: the co-occurence of domestic violence, parental substance misuse, and mental health problems*. Canberra: Australian Institute of Family Studies (2010) [cited 2020 March 25]. Available from: https://aifs.gov.au/cfca/publications/issues-safety-and-wellbeing-children-families-multiple-and-co.

30. Dawe S, Frye S, Best D, Moss D, Atkinson J, Evans C, et al. *Drug use in the family: Impacts and implications for children, ANCD research paper No. 13*. Canberra: Australian National Council on Drugs (2007).

31. Agha MM, Glazier RH, Guttmann A. Relationship between social inequalities and ambulatory care-sensitive hospitalizations persists for up to 9 years among children born in a major Canadian urban center. *Ambul Pediatr* (2007) 7(3):258-62. doi: 10.1016/j.ambp.2007.02.005..

32. Butler DC, Thurecht L, Brown L, Konings P. Social exclusion, deprivation and child health: a spatial analysis of ambulatory care sensitive conditions in children aged 0-4 years in Victoria, Australia. *Soc Sci Med* (2013) 94:9-16. doi: 10.1016/j.socscimed.2013.06.029.

33. Mohanty I, Edvardsson M, Abello A, Eldridge D. Child social exclusion risk and child health outcomes in Australia. *PLoS One* (2016) 11(5):e0154536. doi: 10.1371/journal.pone.0154536.

34. Heukelbach J, Feldmeier H. Scabies. *The Lancet* (2006) 367(9524):1767-74. doi: 10.1016/S0140-6736(06)68772-2.

35. Downe S, Finlayson K, Walsh D, Lavender T. 'Weighing up and balancing out': A meta-synthesis of barriers to antenatal care for marginalised women in high-income countries. *BJOG* (2009) 116(4):518-29. doi: 10.1111/j.1471-0528.2008.02067.x

36. Australian Bureau of Statistics. *Measures of Australia's progress 2013. Cat. No. 1370.0*. Canberra: Australian Bureau of Statistics (2014) [cited 2020 February 25]. Available from: http://www.abs.gov.au/ausstats/abs@.nsf/mf/1370.0.

37. Raines-Milenkov AL. *Health and pregnancy among women with prostitution experience* [Dissertation]. Texas: Univeristy of North Texas (2010).

38. Burnette ML, Lucas E, Ilgen M, Frayne SM, Mayo J, Weitlauf JC. Prevalence and health correlates of prostitution among patients entering treatment for substance use disorders. *Arch Gen Psychiatry* (2008) 65(3):337-44. doi: 10.1001/archpsyc.65.3.337.

39. Lucas K. Transport and social exclusion: where are we now? *Transport Policy.* (2012) 20:105-13. doi: 10.1016/j.tranpol.2012.01.013.

40. Social Exclusion Unit. *Making the connections: Final report on tranport and social exclusion*. London: Office of the Deputy Prime Minister (2003).

41. Kenyon S, Lyons G, Rafferty J. Transport and social exclusion: Investigating the possibility of promoting inclusion through virtual mobility. *Journal of Transport Geography* (2002) 10(3):207-19. doi: 10.1016/S0966-6923(02)00012-1.

42. Currie G, Richardson T, Smyth P, Vella-Brodrick D, Hine J, Lucas K, et al. Investigating links between transport disadvantage, social exclusion and well-being in Melbourne - preliminary results. *Transport Policy* (2009) 16(3):97-105. doi: 10.1016/j.tranpol.2009.02.002.

43. Currie G, Richardson T, Smyth P, Vella-Brodrick D, Hine J, Lucas K, et al. Investigating links between transport disadvantage, social exclusion and well-being in Melbourne – updated results. *Research in Transportation Economics* (2010) 29(1):287-95. doi: doi.org/10.1016/j.retrec.2010.07.036.

44. Manderbacka K, Arffman M, Sund R, Karvonen S. Multiple social disadvantage does it have an effect on amenable mortality: a brief report. *Int J Equity Health* (2014) 13(1):67. doi: 10.1186/s12939-014-0067-5.

45. Putnam RD. The prosperous community: social captial and public life. *The American Prospect* (1993) 13:35-42.

46. Bourdieu P. The forms of capital. In: Richardson R, editor. *Handbook of theory and research for the sociology of education*. New York: Greenwood Press (1986). p.241-58.

47. Uphoff EP, Pickett KE, Cabieses B, Small N, Wright J. A systematic review of the relationships between social capital and socioeconomic inequalities in health: A contribution to understanding the psychosocial pathway of health inequalities. *Int J Equity Health* (2013) 12(1):54. doi: 10.1186/1475-9276-12-54.

48. Distelberg BJ, Martin AvS, Borieux M. A deeper look at the Social Support Index: A multi-dimensional assessment. *Am J Fam Ther* (2014) 42(3):243-56. doi: 10.1080/01926187.2013.837362.

49. Sarason IG, Sarason BR. Social support: Mapping the construct. *J Soc Pers Relat* (2009) 26(1):113-20. doi: doi:10.1177/0265407509105526.

50. Koenig HG, Westlund RE, George LK, Hughes DC, Blazer DG, Hybels C. Abbreviating the Duke Social Support Index for use in chronically ill elderly individuals. *Psychosomatics* (1993) 34(1):61-9. doi: 10.1016/s0033-3182(93)71928-3.

51. Tennant C, Andrews G. A scale to measure the stress of life events. *Aust N Z J Psychiatry* (1976) 10(1):27-32. doi: 10.3109/00048677609159482.

52. Ford RP, Hassall IB, Mitchell EA, Scragg R, Taylor BJ, Allen EM, et al. Life events, social support and the risk of sudden infant death syndrome. *J Child Psychol Psychiatry* (1996) 37(7):835-40.

53. Gilbert R, Widom CS, Browne K, Fergusson D, Webb E, Janson S. Burden and consequences of child maltreatment in high-income countries. *Lancet* (2009) 373(9657):68-81. doi: 10.1016/s0140-6736(08)61706-7.

54. Australian Institute of Health and Welfare. *Children and young people at risk of social exclusion: Links between homelessness, child protection and juvenile justice.* Canberra: Australian Institute of Health and Welfare (2012) [cited 2019 December 8]. Available from: https://www.aihw.gov.au/reports/children-youth/children-and-young-people-at-risk-of-social-exclus/contents/table-of-contents.

55. Cashmore J. The link between child maltreatment and adolescent offending: Systems neglect of adolescents. *Family Matters* (2011) 89:31-41.

56. Lansford JE, Miller-Johnson S, Berlin LJ, Dodge KA, Bates JE, Pettit GS. Early physical abuse and later violent delinquency: A prospective longitudinal study. *Child Maltreatment* (2007) 12(3):233-45. doi: 10.1177/1077559507301841.

57. Stewart A, Livingston M, Dennison S. Transitions and turning points: Examining the links between child maltreatment and juvenile offending. *Child Abuse Negl* (2008) 32(1):51-66. doi: 10.1016/j.chiabu.2007.04.011.
