# Supplementary material for: Applying a Social Exclusion Framework to Explore the Relationship Between Sudden Unexpected Deaths in Infancy (SUDI) and Social Vulnerability
Source: Front Public Health. 2020 Oct 20;8:563573. doi: 10.3389/fpubh.2020.563573 (PMC7606531; doi:10.3389/fpubh.2020.563573)
Supplement: Supplementary file 3 [file Data_Sheet_1.docx]

**Supplementary Figure 1: Scatter plot of correlation between simple summation and family weighted composite vulnerability scores**
